# COMPREHENSIVE HEALTH ASSESSMENT AND MOTIVATION PROGRAM FOR PEOPLE AGING WITH DISABILITIES (CHAMP-D)

**DOI:** 10.1093/geroni/igz038.270

**Published:** 2019-11-08

**Authors:** Patricia C Heyn, James J Carollo

**Affiliations:** 1 University of Colorado Denver Anschutz Medical Campus, Aurora, Colorado, United States; 2 Childrens Hospital Colorado, Aurora, Colorado, United States

## Abstract

We developed a Comprehensive Health Assessment and Motivation program (CHAMP-D) focused on self-health promotion and management for people aging with disabilities. The goal of the CHAMP-D is to enhance healthy lifestyle, self-health management and communications with the care team by a person-centered Health Passport tool. All study participants underwent comprehensive health, physical, and blood laboratory evaluation. CHAMP-D results were formatted into an easy to follow health passport. A follow-up survey evaluated the CHAMP-D on 59 respondents and 77% found the recommendations to be achievable and reported improved quality of life (QoL). Other improvements were noted in in self-care (54%), physical activity (49%), and diet (24%). 51% of the participants shared the Health Passport with their PCP. Overall, the CHAMP-D had a positive impact on participants’ self-reported health, self-care, and wellbeing. Empowering individuals with disabilities to take an active role in self-managing their health is feasible and can impact their QoL.

